# *Bacillus cereus* as a Major Cause of Discarded Pasteurized Human Banked Milk: A Single Human Milk Bank Experience

**DOI:** 10.3390/foods10122955

**Published:** 2021-12-01

**Authors:** Miroslava Jandová, Pavel Měřička, Michaela Fišerová, Aleš Landfeld, Pavla Paterová, Lenka Hobzová, Eva Jarkovská, Marian Kacerovský, Milan Houška

**Affiliations:** 1 Tissue Bank, University Hospital Hradec Králové, 500 05 Hradec Králové, Czech Republic; pavel.mericka@fnhk.cz (P.M.); michaela.fiserova@fnhk.cz (M.F.); 2 Department of Histology and Embryology, Faculty of Medicine in Hradec Králové, Charles University, 500 03 Hradec Králové, Czech Republic; 3 Food Research Institute Prague, 102 00 Prague, Czech Republic; ales.landfeld@vupp.cz (A.L.); milan.houska@vupp.cz (M.H.); 4 Department of Clinical Microbiology, University Hospital and Faculty of Medicine in Hradec Králové, Charles University, 500 05 Hradec Králové, Czech Republic; pavla.paterova@fnhk.cz; 5 Department of Hospital Hygiene, University Hospital Hradec Králové, 500 05 Hradec Králové, Czech Republic; lenka.hobzova@fnhk.cz; 6 Department of Pediatrics, University Hospital Hradec Králové, 500 05 Hradec Králové, Czech Republic; eva.jarkovska@fnhk.cz; 7 Department of Obstetrics and Gynecology, University Hospital Hradec Králové, 500 05 Hradec Králové, Czech Republic; marian.kacerovsky@fnhk.cz

**Keywords:** *Bacillus cereus*, human milk, pasteurization, risk assessment

## Abstract

A systematic study, performed from 2017–2020 looked at the rate of positive post-pasteurization *B. cereus* findings, the quantity of *B. cereus* in pasteurized banked human milk (PBM), and the rate of *B. cereus* toxicogenic isolates from PBM. During the study period, 6815.71 L (30,943 tested bottles) of PBM were tested, with an average amount per year of 1703.93 L (7736 tested bottles). The PBM discard rate per year due to bacterial contamination varied between 8.7–10.0% and contamination with *B. cereus* was the most frequent reason. The total number of *B. cereus* positive tests was 2739 and the proportion of its positivity from all positive tests was between 56.7–66.6%. The prevalence of *B. cereus* positive tests rose significantly in the summer months. The production of enterotoxin was found in 3 of the 20 tested samples (15.0%). The *B. cereus* CFU-quantities in the PBM were below 10 CFU/mL in 80% of cases (16 of 20 samples tested). The quantitative data can be used in the risk assessment of cold storage of PBM at temperatures above zero and manipulation of PBM prior to its administration.

## 1. Introduction

*B. cereus* is a motile aerobic or facultatively anaerobic, spore-forming Gram-positive bacterium. It is found in the environment, air, dust, and water and is a common contaminant of food [1], including milk formulas [2,3,4,5]. Some of its strains are toxicogenic [2,6,7]. *B. cereus* is responsible for two types of food poisoning: diarrheal syndrome and emetic syndrome [7,8]. *B. cereus* is an opportunistic human pathogen associated with local and systemic infections in immunosuppressed patients. Premature infants are highly susceptible to infection due to their poorly developed immune system and prolonged invasive procedures, such as mechanical ventilation or catheterization [9,10]. Infections of the bloodstream, lungs, central nervous system [1,11,12,13,14], and intestinal tract [2] have been described, some with fatal outcomes [9,15]. The bacterium is resistant to standard cleaning procedures used in the food industry and hospitals as well to pasteurization [7,16,17,18,19,20]. Decousser et al. first described the presence of pathogenic strains of *B. cereus* in pasteurized banked milk [2]. In recent years several studies described *B. cereus* as a frequent cause of PBM discard after Holder pasteurization that is used in most Human Milk Banks [20,21,22]. Experiments were performed with other pasteurization regimens, no evidence of their better efficiency on *B. cereus* was presented, however [22]. High-pressure inactivation [23] seems to be a promising method for the future. Since outbreaks of *B. cereus* infection occur in neonatal intensive care units, PBM becomes a suspect source of infection [16,24]. However, PBM was never confirmed as a source of *B. cereus* infection in newborns. Nevertheless, especially the latter case from August/September 2016 [24] led to increased interest in detecting *B. cereus* in PBM and in identifying potential sources of *B. cereus* infection in newborns. Fournier et al. analyzed nine cases of *B. cereus* bacteremia that occurred in neonatal intensive care units of five French hospitals from August till December 2016 and reviewed the corrective actions made in the hospitals and in the Human Milk Bank [24]. Glasset et al. [19] presented the results of the 5-year retrospective study of *B. cereus* infections detected in nine hospitals from different regions of France in the years 2008–2012. *B. cereus* was found in cultures taken from 39 patients, 41% of them were newborns. The authors point out the clinical significance of positive *B. cereus* findings in the environment of hospitals, which was generally underestimated in the past. Recently Cormontagne et al. published an extensive review summarizing the spectrum of possible *B. cereus* infections in premature newborns, the possible sources of *B. cereus* in the environment of hospitals including contaminated medical devices used in pediatric intensive care units, as well as the data on post-pasteurization contamination of human milk in French Human Milk Banks [20]. In this review, *B. cereus* was reported to be the leading cause of PBM discard that varied between 13.9% and 21.2% in the years 2016 to 2019. *B. cereus* was found in 80 to 90% of discarded batches [20]. Similarly to the above-cited authors, we have paid particular attention to the presence of *B. cereus* in PBM processed in our Milk Bank since the end of 2016. In 2017 we introduced an improved method of detection of all strains surviving pasteurization using the MALDI mass spectrometry method. This paper presents the results of a 4-year systematic study performed from 2017 to 2020 that focused exclusively on human milk banking issues: 1. Frequency of all microbial strains surviving pasteurization after the introduction of the MALDI method; 2. The proportion of PBM contamination caused by the *Bacillus* genus and particularly by *B. cereus* of the total PBM contamination and its seasonal prevalence; 3. The quantity of *B. cereus* in milk discarded due to *B. cereus* positivity; 4. The frequency of *B. cereus* strains producing enterotoxin in isolates from *B. cereus* positive milk. The post-pasteurization *B. cereus* quantity and toxigenicity were investigated in two prospective studies performed in 2020.

## 2. Materials and Methods

### 2.1. The Human Milk Bank Quality Management System

In 2004, we implemented a Hazard Analysis and Critical Control Points system (HACCP) [25,26], which has been regularly updated. This system is compliant with the requirements of the Regulation of the European Parliament No. 852/2004, Council on Hygiene of Food and Regulation of the European Commission No. 2073/2005, and the Decree of Ministry of Health of the Czech Republic No. 602/2006 Coll. A first version of HACCP handbook was written in the same year. Besides the analysis of physical, chemical and especially microbiological risk during the collection, processing, storage, and distribution of PBM this handbook implemented the basic principles of good manufacturing practice, such as the use of regularly validated equipment, continuous monitoring and recording of critical parameters, prevention of secondary contamination during processing by the use of a laminar flow cabinet and release of each batch for clinical application by a qualified person. A predictive mathematical model was used to assess the bacteriological risk during the manipulation of pasteurized human milk [25]. The HACCP handbook for our milk bank includes a schematic description of the manufacturing process with marked control points. The last update of the handbook in 2016 included improvements in bacteriological diagnostics using the MALDI method, leading to a more precise microbiological risk assessment.

### 2.2. Human Milk Collection and Receipt by the Milk Bank

Milk collection was performed on mothers hospitalized with their newborns requiring intensive care in the pediatric department or the homes of external milk donors. Milk was expressed by hand or with a breast pump. Donors were instructed by nurses from the pediatric department or from the Human Milk Bank (in the case of external donors) on compliance with the rules of hygienic collection [27] (hands washed, all components of the breast pump cleaned and disinfected). Milk was collected into 250 mL sterilized glass bottles distributed by the Human Milk Bank. Donors could bring either native fresh milk cooled to +4°C–+10°C or frozen milk. Upon receipt, the nurses at the Human Milk Bank made a visual inspection of the integrity of the glass bottles and stoppers. In the case of frozen milk, the milk had to be visibly frozen. The nurses also checked donors’ documentation.

### 2.3. Pre-Pasteurization Risk Assessment and Input Microbiological Control

For fresh milk, an initial quantitative microbiological assessment was conducted before pasteurization. The microbiological assessment was a check for the presence of aerobic bacteria and fungi in the milk. All microbiological testing was performed in the Department of Clinical Microbiology at the University Hospital and Faculty of Medicine in Hradec Králové. The quantitative pre-pasteurization evaluation was done according to the European Pharmacopoeia (EPh) Chapter 2.6.12: Microbiological testing of non-sterile products and water by direct inoculation on agar [28]. Five-dilution rows (dilution 1:10) were prepared in sodium chloride solution based on an expected bioburden of 103–105 CFU/mL. Each dilution of milk was inoculated on Columbia agar (Oxoid Ltd., Hampshire, UK). All plates were placed in an incubator (Memmert GmbH + Co. KG, Schwabach, Germany) for 18–24 h in an ambient atmosphere at 35 °C ± 2 °C. The following day all isolates were identified using MALDI-TOF MS (Bruker Daltonics, Hamburg, Germany). The colony count was determined based on the growth of microbes in the final dilution.

We used European and Australian standards as the criteria for the pre-pasteurization discard of milk (Table 1) [26].

### 2.4. Processing and Post-Pasteurization Microbiological Evaluation

**Figure 1:**
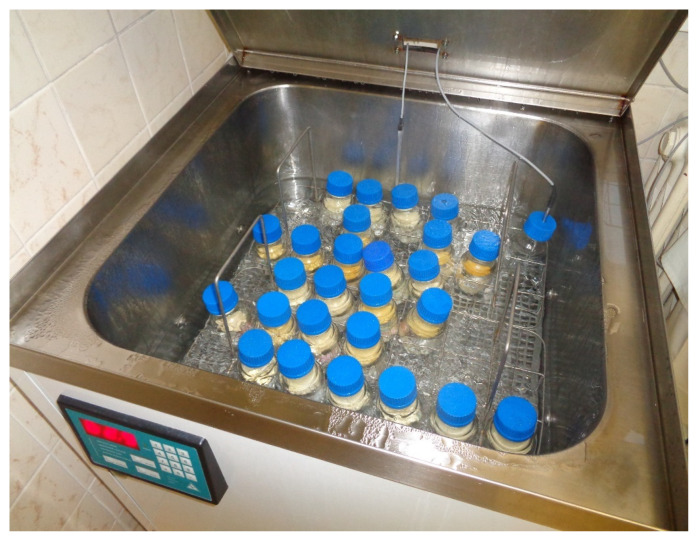
Bottles with collected milk in the warming section of the pasteurization device. The temperature is monitored and recorded inside the water bath and in the bottle containing the milk analog.

After testing, the milk was processed on the same day or after separate intermediate storage in a freezer (Liebherr GG 5210, Liebherr Hausgeräte, Ochsenhausen, Germany) operating from −25 °C to −27 °C. Bottles with fresh or frozen milk were put into a water bath of the warming section of the computer-assisted pasteurization equipment, where the milk was thawed and then pasteurized. The standard Holder pasteurization (62.5 °C for 30 min) followed by chilling was used as recommended by EMBA (European Milk Bank Association) standards [26]. For the warming phase, we used the BW-50 programmable water bath (ALFAMEDIC Ltd., Lišov, Czech Republic), in which the pasteurization process was regulated according to the temperature achieved inside a reference bottle containing a milk analog (Figure 1).

**Figure 2:**
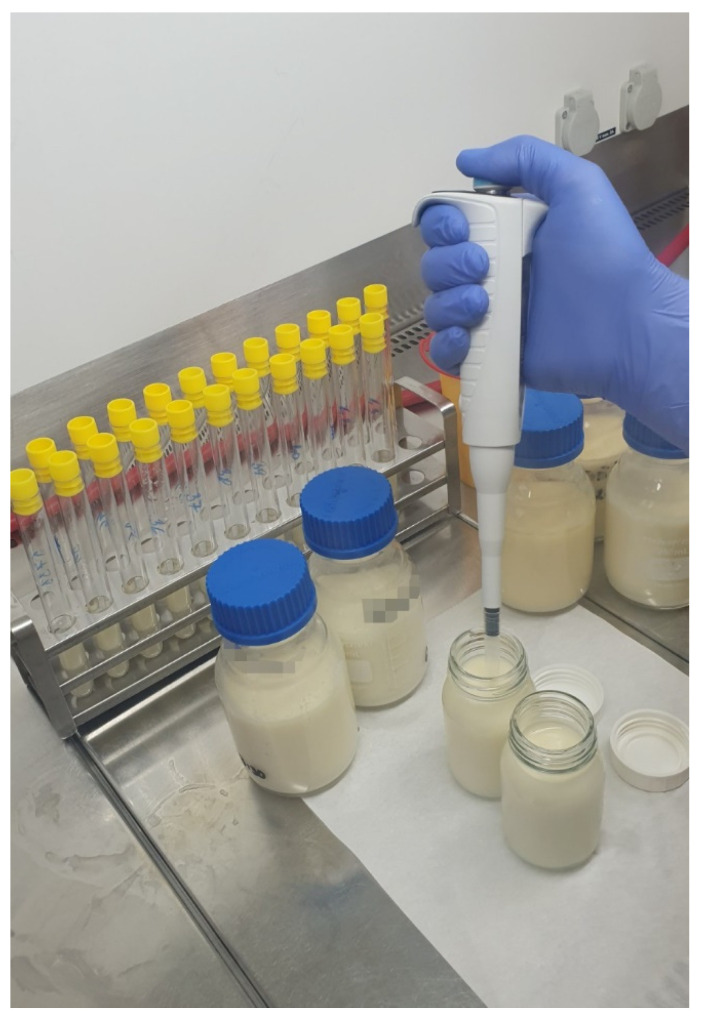
Collection of the milk samples for qualitative bacteriological analysis.

Immediately after pasteurization, the bottles were moved to a programmable chilling water bath (BW-50.1) of the same producer as mentioned above and the milk was chilled to +15 °C ± 0.5 °C. The temperature was again measured in a reference bottle, and chilling was automatically stopped at +15 °C. After chilling, the bottles were removed and placed into a laminar flow cabinet (Alpina Bio 130, Alpina, Konin, Poland). For qualitative microbiological evaluation, 1 mL of milk from each bottle was inoculated into 5 mL of thioglycollate broth (LMS, Ústí nad Labem, Czech Republic) (Figure 2).

**Figure 3:**
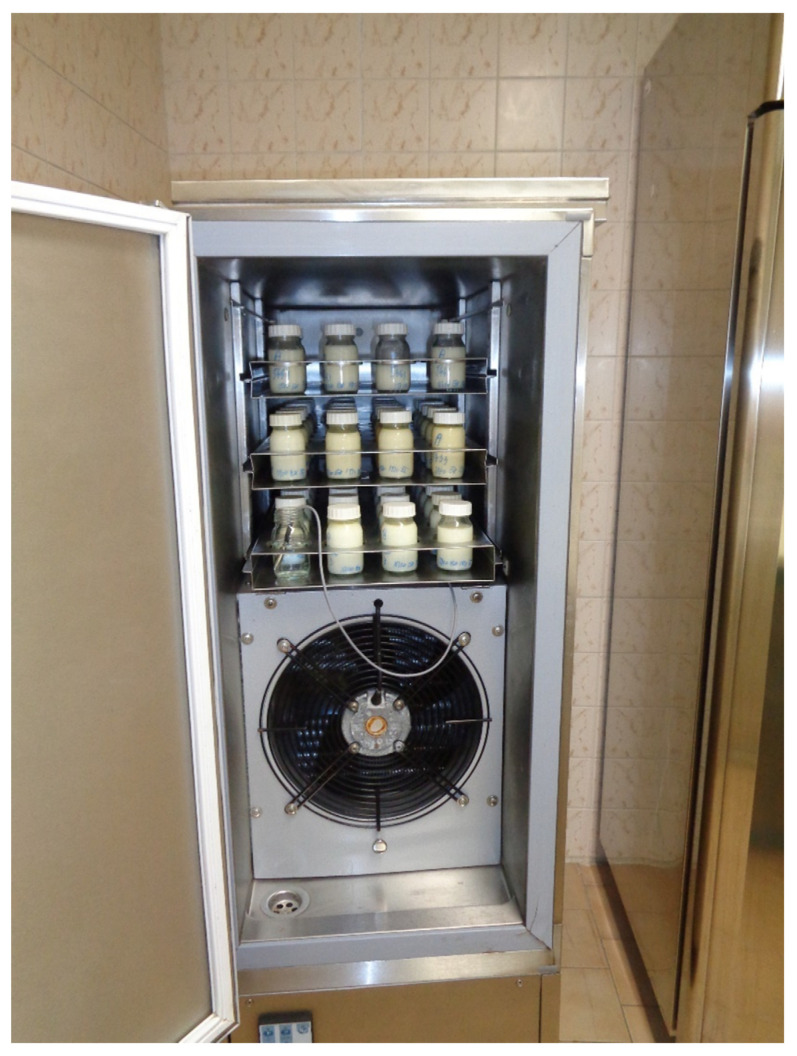
Distribution bottles in the blast freezer. The temperature of the air and inside the bottle containing the milk analog is recorded.

The milk was divided into 100- or 50-mL sterile distribution bottles and sampling was performed (Figure 2). Then the bottles were rapidly frozen to −16 °C in a blast freezer (CZM 20/8V, Horákové Brothers, Lužec nad Vltavou, Czech Republic) (Figure 3).

The process takes 1–2 h, depending on the number of bottles in a pasteurization batch. The frozen bottles are removed and stored separately in a quarantine freezer. The temperature data for pasteurization, chilling and freezing for each batch were recorded by the system (Netcom and Read 95, Regucon, Ltd., Prague, Czech Republic) and stored in electronic and printed form. These records were reviewed by qualified person of the Human Milk Bank before the release of each batch for clinical use.

All equipment used for pasteurization, freezing and storage of human milk was regularly validated by Kalist, Ltd., Holešov, Czech Republic. The parameters of the environment in the Human Milk Bank premises, temperature and relative humidity were continuously monitored and recorded by the same system as mentioned above.

All vials with inoculated thioglycollate broth were incubated in the dark for 18–24 h in an ambient atmosphere at 35 °C ± 2 °C (at the Department of Clinical Microbiology) and then inoculated on Columbia agar (Oxoid, Ltd., Hampshire, UK). All plates were incubated again for 18–24 h in an ambient atmosphere at 35 °C ± 2 °C. The following day, colonies of microbes were found, and all isolates were identified using MALDI-TOF MS (Bruker Daltonics, Hamburg, Germany).

**Figure 4:**
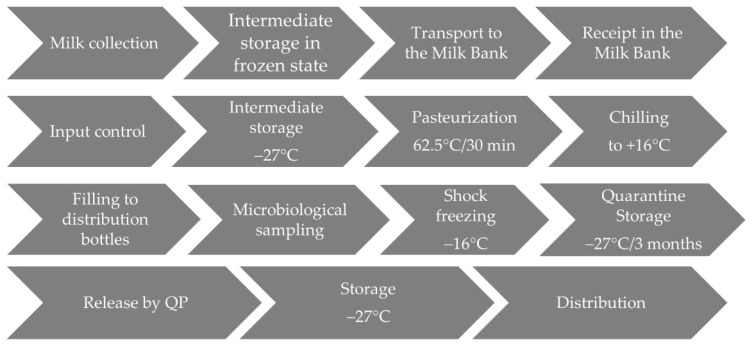
Diagram of the manufacturing and control processes.

Only bottles with negative post-pasteurization microbiological results were released for clinical use and transferred to a dispensing freezer (Liebherr GG 5210, Liebherr Hausgeräte, Ochsenhausen, Germany) operating at a temperature from −25 °C to −27 °C). The shelf life of frozen milk stored at this temperature was three months. A schematic description of the manufacturing and control processes is presented in Figure 4.

### 2.5. Design of the Experiment

**Figure 5:**
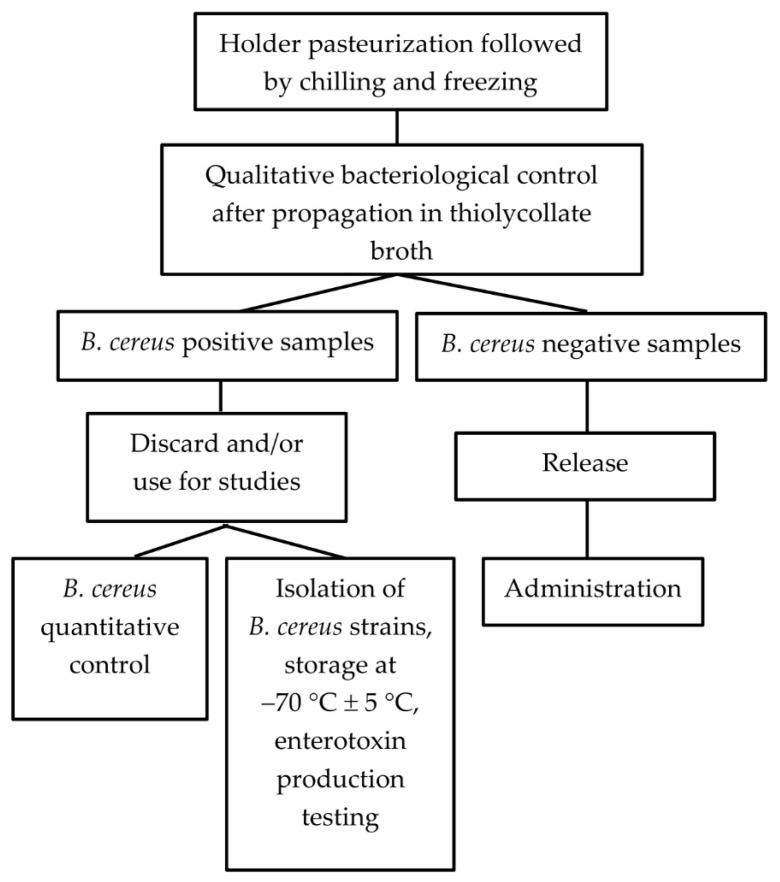
The design of the study.

The design of the study is shown schematically in Figure 5, and a detailed description of the methodology is presented in Section 2.5.1, Section 2.5.2, Section 2.5.3 and Section 2.5.4

#### 2.5.1. Analysis of the Presence of *B. cereus* in PBM

The first part of our study was based on a retrospective analysis of results of 30,943 tests performed during the four-year study period as a part of routine qualitative bacteriological control described in Section 2.4.

#### 2.5.2. Seasonal Prevalence of *B. cereus*

To determine whether *B. cereus* in PBM would change significantly during the warmer months of the year, we compared its frequency in the cold and warm periods of the tested years. To compensate for considerable temperature deviations from the norm in the spring months of 2018 and 2019, we chose April to September as the warmer months and October to March as the colder months. Tables S1–S3 showing the mean daily temperatures in April, May, and September and deviations from the norm in the observed period reported by INFOMET [29], have been added as supplementary data. Categorical variables were compared using the Fisher exact test and presented as numbers (%). Differences were considered significant at *p* < 0.05. All *p*-values were obtained using two-tailed tests. All statistical analyses were performed using GraphPad Prism version 8.4.3.

In the second part of our investigations, two separate prospective studies were performed. In 20 samples originating from 20 donors, revealing a post-pasteurization *B. cereus* positivity quantitative evaluation was made as described in Part Section 2.5.3. In another 20 samples, the *B. cereus* strains were isolated and stored in a frozen state for enterotoxin production (diarrheal type) testing (Part Section 2.5.4).

#### 2.5.3. Quantitative Post-Pasteurization Bacteriological Evaluation of *B. cereus* Positive Milk

Quantitative determination was carried out per European Pharmacopoeia (EPh) Chapter 2.6.12: Microbiological testing of non-sterile products and water by direct inoculation on agar [28]. 500 μL of PBM was inoculated directly on two Columbia agars (Oxoid), the agars were further incubated at an ambient atmosphere of 35 ± 2 °C. After 18–24 and 48 h count of CFU (colony forming unit) per mL for each milk sample and time was determined.

#### 2.5.4. Enterotoxin Production Assessment

This prospective study collected *B. cereus* strains from pasteurized milk processed in 2020, one sample per donor. The strains were stored frozen in the cryobank at −70 °C ± 5 °C (I-TEST Plus, Hradec Králové, Czech Republic) and revitalized according to the manufacturer’s instructions. Enterotoxin production was determined using Oxoid^TM^ BCET-RPLA Toxin Detection Kit (Thermo Fisher Scientific, Waltham, Massachusetts, USA) in V-well microtiter plates. If *B. cereus* enterotoxins were present, agglutination occurred due to the formation of a lattice structure [30].

## 3. Results

### 3.1. Basic Characteristic of Human Milk Bank Donation and Processing Activities

The total amount of collected milk during the study period was 6815.71 L (30,943 bottles), with an average of 1703.93 L per year. There has been an increasing trend in the total amount of collected milk over the last four years (Table 2); most of the milk came from mothers hospitalized in the Pediatric department with their newborns.

### 3.2. Results of the Pre-Pasteurization (Input) Bacteriological Control

Table 3 shows the results of the pre-pasteurization (input) microbiological evaluation.

### 3.3. Results of Post-Pasteurization Control

During the study period, 30,943 tests were performed after pasteurization. Positive microbiological findings were detected in 2739 (8.85%) cases. Table 4 presents the total post-pasteurization PBM discard rate over the last four-year study. The data show a steady discard rate due to microbiological findings over the period.

#### The Spectrum of Bacterial Isolates

**Figure 6:**
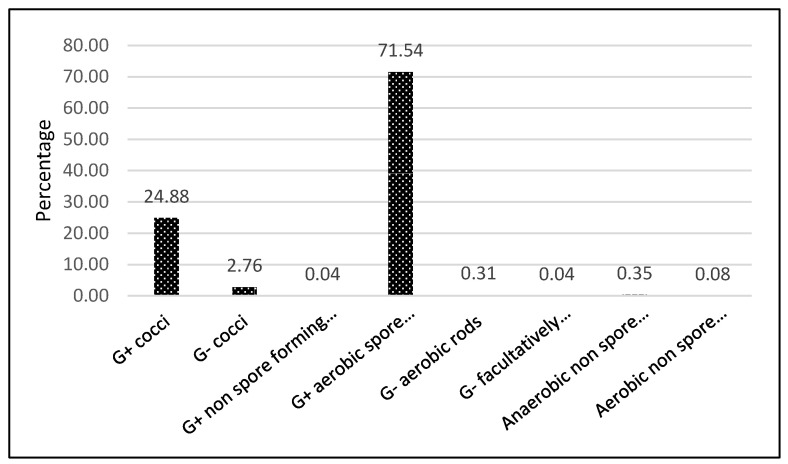
Frequency [%] of microbial groups in PBM (2017–2020).

The spectrum of bacterial isolates is shown in Figure 6. Aerobic Gram-positive spore-forming bacteria dominated the positive post-pasteurization findings. The most common group was Gram-positive spore-forming rods (71.54%), including *B. cereus* (63.99%). The second most dangerous bacterium in milk, *Staphylococcus aureus* (Gram-positive cocci), was found in 30 tests, but only caused the elimination of only 1.10% PBM. No yeasts or molds were found.

**Figure 7:**
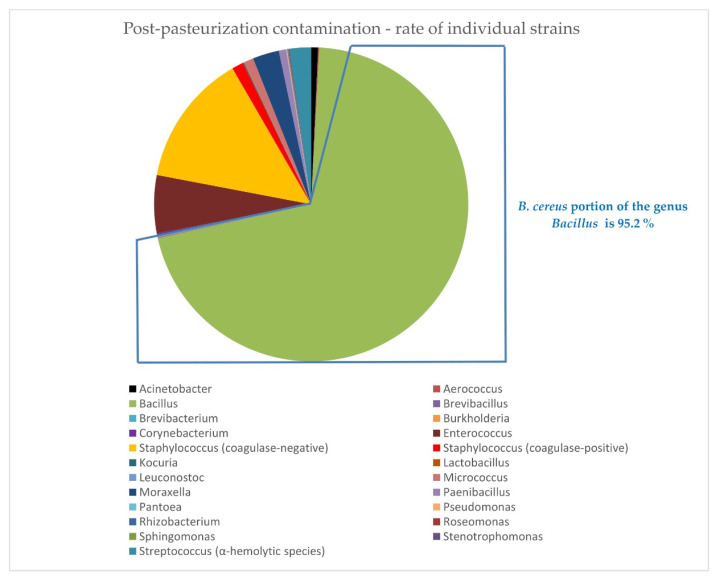
Post-pasteurization contamination shows percentage contamination by individual strains.

The proportion of *B. cereus* is presented in Figure 7, as well as the relative frequency of individual strains surviving pasteurization in Table 5.

### 3.4. Assessment of the Seasonal Prevalence of B. cereus

**Figure 8:**
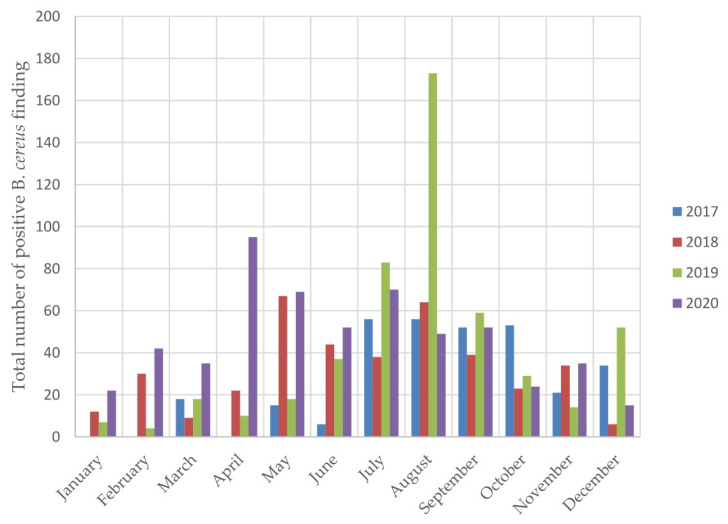
The frequency of positive *B. cereus* findings in individual months.

The results shown in Table 6 indicate that the seasonal prevalence of *B. cereus* was confirmed in all monitored years. The prevalence of *B. cereus* in warm months was significantly higher than in the colder months of the same year. Figure 8 shows the frequency of positive *B. cereus* findings in individual months.

### 3.5. Results of the B. cereus Quantitative Post-Pasteurization Evaluation

The results are presented in Table 7. In 80% of cases, a quantity lower than 10 CFU/mL was found.

### 3.6. Results of the Enterotoxin Production Assessment

The production of diarrheal enterotoxin, assessed by reversed passive latex agglutination, was found in 3 of 20 (15.0%) *B. cereus* isolates from PBM.

## 4. Discussion

*B. cereus* survival in pasteurized milk is well established [2,31]; studies examining the frequency of positive *B. cereus* findings after Holder pasteurization, which is used by most Human Milk Banks and as recommended by EMBA, are only now starting to appear [18,19,20]. Simultaneously research into *B. cereus* toxigenicity has been intensified [20,32]. It has been caused by the fact that in the past, the main focus was *S. aureus*, *enterococci*, and *coagulase-negative staphylococci* in PBM [25], while detailed diagnostics of the genus *Bacillus* was not routinely performed. The positive findings remained hidden in the group evaluated as *Bacillus* sp. Using the MALDI method, Mullié et al. (2018) described the *Bacillus* genus as the leading cause of post-pasteurization non-compliance, followed by *coagulase-negative Staphylococci*. Recently Cormontagne et al. identified *B. cereus* as the leading cause of post-pasteurization PBM discard at Human Milk Banks using similar processing and control procedures as those described in this study [20]. The study of Cormontagne et al. also examined seasonal fluctuation of *B. cereus* prevalence and described a peak in the summer months.

Our study also found that *B. cereus* was a major cause of PBM discard (Figure 7); its proportion was, however, lower than reported by Cormontagne. Moreover we studied in detail the differences in the month-to-month prevalence of *B. cereus* positivity during the year (Figure 8, Table 6) and found a statistically significant increase in *B. cereus* prevalence in the warmer months (Table 6). In our case the warmer months included summer months, as well as spring months, i.e., April and May of the observational years, when temperatures were well above average for the Czech Republic (Tables S1–S3 in the supplementary data). Moreover, 2018 was the warmest year of the last 60 years, which was followed by 2019. This undoubtedly reflects the influence of climate changes and points to the need to implement the technical and organizational measures required for careful maintenance of the cold chain during the whole process of collection, transport, storage and distribution of PBM, including strict adherence to the instructions issued by Human Milk Banks regarding the proper use of PBM in pediatric wards.

Better diagnostics has led to an enlargement of the spectrum of microbes capable of surviving pasteurization (Figure 7, Table 5). In addition to the pathogenic or potentially pathogenic genera identified in our previous studies [25,33], *Burkholderia, Kocuria, Moraxella, Roseomonas, Sphingomonas* and *Stenotrophomonas* were identified in this study (Figure 7, Table 5). We encountered similar genera described by others who used the MALDI method [31], e.g., *Moraxella* spp., *Stenotrophomonas* spp., and *Acinetobacter* spp.

There is no consensus about the acceptable bacteriological contamination of PBM. While some standards permit contamination (all strains) lower than 10 CFU/mL (Italy, Sweden, UK), others do not tolerate any post-pasteurization contamination (France, Australia, USA) [26]. The post-pasteurization quantities of *B. cereus* CFU found in this study were within the 10 CFU/mL limit in most (80%) cases (Table 7). However, the current trend is not to permit any *B. cereus* contamination after pasteurization [20]; in which case only 20% of samples presented in Table 7 were *B. cereus* negative and/or had contamination below the detection limit. Together with the low prevalence of toxicogenic *B. cereus* isolates (15%), we regard this finding as good evidence of the efficiency of our current pasteurization process. On the other hand, occasional findings (5%) of 100 CFU/mL show that even when following EMBA recommendations for pasteurization and GMP principles, the routine post-pasteurization bacteriological controls we performed are fully justified to assure the safety of the product.

Our total PBM discard rate due to positive post-pasteurization findings fluctuated between 8.6% and 10.5% (Table 4) and was in the middle of the interval of the total discard rate found by Dewitte et al. in a survey performed in six major French Human Milk Banks (4–16%) [34]. It was also lower than the discard rate reported in a recent review by Cormontagne [20]. This might be caused by the fact that we perform a complete post-pasteurization control of each bottle, thereby eliminating the need to discard whole PBM batches that consist of large quantities of milk pooled from several human milk donors. Our technology also makes it possible to trace milk from donors to premature newborns fed with PBM products; we regard traceability as an important principle of our Quality Assurance System. This aspect of safety assurance was pointed out also by Fournier et al. [24].

Another cause of our relatively low PBM discard rate might be the fact that in our practice the majority of collected milk is frozen immediately after collection. The results of input microbiological control performed in the rare cases when milk is referred to the bank in the liquid state (Table 3) reveal good compliance with the generally accepted limits (Table 1). Our results show the importance of careful analyses of the prevalence of different bacteriological strains that show higher resistance to the pasteurization process as a part of the implementation of the HACCP system, which is an important part of the Quality Management System recommended by EMBA [26]. The spectrum of strains presented in this study may be hospital and/or pediatric department-specific and may change over time; nevertheless, similar spectra of surviving microbes have been reported also by other authors [2,31].

It is clear that when using standard Holder pasteurization, bacterial spores of bacteria are capable of surviving the pasteurization process, meaning that any further lowering of the PBM discard rate may not be possible. Changing technology to the innovative pasteurization procedures recently described [20,22] might improve discard rates, it would require a long period of experimental verification. High-pressure inactivation, which Demazeau described as being effective against spores [23], is regarded by us to be a promising method for the future; as our group also found the technique effective on bacteria present in human and cow milk in the past [33,35].

In our system, all PBM is distributed in the frozen state which eliminates the possibility of growth of vegetative *B. cereus* forms surviving pasteurization, which can occur if PBM is stored at refrigeration temperatures [8]. Since pasteurization impairs the natural antibacterial properties of human milk [36], it creates conditions suitable for the multiplication of microbes that can be more rapid than in case of storage of liquid raw human milk above zero degrees. Moreover, *B. cereus* is capable of growing even at temperatures near zero. These characteristics of *B. cereus* must be considered during risk assessment of cold storage of PBM as well as storage and use of PMB on the pediatric wards. There are growth models available for many microbes surviving pasteurization [25,35,37], which are suitable tools for properly assessing this risk.

## 5. Conclusions

In Human Milk Banks using the standard Holder pasteurization process, *B. cereus* may substantially contribute to the rate of PBM discard performed based on the post-pasteurization bacteriological control. In this study, *B. cereus* was the leading cause of PBM discard, and the discard rate significantly increased during the warmer months of the year (April to September). *B. cereus* PBM CFU-quantities described in this study were mostly below 10 CFU/mL; nevertheless, occasional findings of higher quantities fully justified the routine post-pasteurization bacteriological control as recommended by the EMBA. Post-pasteurization survivability of *B. cereus* must be taken into account when assessing the risk of cold storage of PBM at temperatures above zero and when assessing the risk of storage and use of PBM in the pediatric wards prior to administration to newborns.

Regular evaluations of the spectrum of strains surviving pasteurization, including *B. cereus*, and monitoring changes and trends should be a part of the Quality Management System of Human Milk Banks.

The post-pasteurization discard rate of 8.23–10.0% described in this study is acceptable and lower than reported from other Human Milk Banks using similar processing and control methods. According to the authors´ opinions, further lowering of discard rates is scarcely possible without changes in decontamination technologies. The authors regard high-pressure inactivation to be a promising method for the future.

## Figures and Tables

**Table 1:** Criteria for the pre-pasteurization discard of milk.

Standards	Exclusion Criteria
French legislation	Total bacteria ≥ 10^5^ CFU/mL
Italian guidelines	*Staphylococcus aureus* > 10^4^ CFU/mL
French legislation	*Staphylococcus coagulase-positive* ≥ 10^4^ CFU/mL
Australian guidelines	Any *enterobacteriaceae*, *enterococci*, or potential pathogens capable of producing heat-stable enterotoxins

**Table 2:** The total amount of collected milk and the sources, i.e., external or hospitalized donors.

Year	Total Amount of Collected Milk (L)	Milk from External Donors [%]	Milk from Hospitalized Donors [%]
2017	1630.05	16.68	83.32
2018	1454.65	6.35	93.65
2019	1597.30	14.45	85.55
2020	2133.71	36.90	63.10

**Table 3:** Results of the input microbiological evaluation.

Year	Total Number of Input Tests	Total Bacteria ≥ 10^5^ CFU/mL	*Staphylococcus aureus* > 10^4^ CFU/mL
2017	11	1	0
2018	29	0	1
2019	22	0	0
2020	10	0	1

**Table 4:** Percentage of discarded PBM due to input and post-pasteurization positive microbial findings.

Year	Input Control [%]	Post-Pasteurization Control [%]	Total [%]
2017	0.28	8.62	8.90
2018	0.43	8.23	8.66
2019	0.00	10.00	10.00
2020	0.10	9.27	9.37

**Table 5:** Results of post-pasteurization microbiological evaluation—the relative frequency of individual strains.

Genus	(%)	Genus	(%)
*Acinetobacter*	0.71	*Leuconostoc*	0.04
*Aerococcus*	0.12	*Micrococcus*	0.94
*Bacillus*	70.63	*Moraxella*	2.71
*Brevibacillus*	0.08	*Paenibacillus*	0.75
*Brevibacterium*	0.04	*Pantoea*	0.04
*Burkholderia*	0.04	*Pseudomonas*	0.08
*Corynebacterium*	0.35	*Rhizobacterium*	0.04
*Enterococcus*	5.94	*Roseomonas*	0.04
*Staphylococcus (coagulase-negative)*	13.65	*Sphingomonas*	0.04
*Staphylococcus (coagulase-positive)*	1.18	*Stenotrophomonas*	0.12
*Kocuria*	0.08	*Streptococcus (α-hemolytic species)*	2.32
*Lactobacillus*	0.08		

**Table 6:** Seasonal prevalence of *B. cereus*.

		Prevalence (%)		
Year	Period	*B. cereus*	Other Microbes	*p* Value
2017	April–September	60	44	*p* < 0.0001
	October–March	40	56	
2018	April–September	71	53	*p* < 0.0001
	October–March	29	47	
2019	April–September	75	60	*p* < 0.0001
	October–March	25	40	
2020	April–September	68	55	*p* = 0.0004
	October–March	32	45	

**Table 7:** Results of the quantitative *B. cereus* post-pasteurization evaluation.

CFU/mL Range	Frequency	Relative Frequency (%)	Cumulative Relative Frequency (%)
Negative	4	20	20
1–5	10	50	70
6–10	2	10	80
11–15	1	5	85
16–20	0	0	85
21–25	1	5	90
26–30	0	0	90
31–35	0	0	90
36–40	0	0	90
41–45	0	0	90
46–50	0	0	90
51–99	1	5	95
100	1	5	100
Total	20	xxx	xxx

## Data Availability

Not applicable.

## Supplementary Materials

The following are available online at https://www.mdpi.com/article/10.3390/foods10122955/s1, Table S1: Temperatures in April, Klementinum, Prague, Table S2: Temperatures in May 2017–2020, Klementinum, Prague, Table S3: Temperatures in September 2017–2020, Klementinum, Prague.
