# Supplementary material for: Bacillus cereus as a Major Cause of Discarded Pasteurized Human Banked Milk: A Single Human Milk Bank Experience
Source: Foods. 2021 Dec 1;10(12):2955. doi: 10.3390/foods10122955 (PMC8700665; doi:10.3390/foods10122955)
Supplement: Supplementary file 1 [file foods-10-02955-s001.zip › foods-1428760-supplementary.pdf]

Table S1. Suppl. Temperatures in April, Klementinum, Prague

Year	Mean (°C)	Deviation from norm (°C)*	Maximum (°C)	Minimum (°C)
2017	10.0	+ 0.4	16.0	3.5
2018	15.6	+ 6.1	21.3	6.7
2019	12.4	+ 2.6	20.5	4.6
2020	12.6	+ 2.8	18.7	4.0

Table S2. Suppl. Temperatures in May 2017 - 2020, Klementinum, Prague

Year	Mean temperature of the month °C	Deviation from norm °C*	Maximal mean day temperature °C	Minimal mean day temperatuire °C
2017	17.1	+2.3	25.3	6.9
2018	19.6	+3.7	26.0	13.3
2019	13.8	-2.1	20.0	5.9
2020	14.3	-1.6	18.9	7.8

Table S3. Suppl. Temperatures in September 2017 – 2020, Klementinum, Prague

Year	Mean (°C)	Deviation from norm (°C)*	Maximum (°C)	Miínimum (°C)
2017	14.8	- 0.4	18.0	11.5
2018	17.5	+ 2.3	22.7	9.4
2019	16.4	+ 0.8	22.0	11.5
2020	17.3	+ 1.7	23.9	8.6

*Deviation from norm calculated from mean temperatures in the period 1981- 2010. Positive deviations are marked red.
